# Colorado Veterinarians Seek State Recognition

**Published:** 1897-01

**Authors:** 


### COLORADO VETERINARIANS SEEK STATE RECOGNITION.

THE veterinarians of the "Gold-hunters' State," fully alive to their needs and the dangers of delay, have presented to their State Legislature one of the best bills yet offered for regulating the practice of veterinary science. Learning of the danger of their State becoming the haven of refuge for the illiterate sharks and pretenders driven from other States where laws now prevail, they desire to be forearmed in this much-needed direction. Colorado has not as yet suffered, like many of the Eastern States, from a horde of these quacks and impostors, and therefore the citizens of that State can scarcely realize the extent and force of their depredations, and we trust that the legislature will pass this meesnre and extend to those who are endeavoring to render them scientific care and protection for their live-stock industry a just measure of recognition and support. The bill has several very excellent features about it; among others, that all registrations under the law will be made to the Board and not to a county officer, whose interpretation of the spirit and intent of such acts wholly depends upon whether he receives a salary or is dependent upon the fees of his office. A second good feature is the fee of ten dollars for resident non-graduates registering under one of the provisions of the Act. A fee of one dollar in Pennsylvania led to several hundred registrations under the original Act when a proper fee would have proved a barrier. The recognition of different schools of the Board assures fairness to all and adds strength to the measure. Every veterinary college, every association, and every member of the profession throughout the land should assist their colleagues in placing this measure on the statute-books of Colorado. It will be a boon to the owners of live-stock, and will afford the citizens of that State an intelligent and deserved protection for their investments, which alone can insure them against needless and disastrous losses where combined ignorance and knavery prevail in scientific fields.

Michigan and New Hampshire will ask at the hands of their lawmakers greater safeguards in better legislation for the supervision of the practice of veterinary science in these States, and we are sure that the people of these States, as well as of North Carolina, another commonwealth seeking similar favor and support, will not regret the passage of any law that shall lead to a higher degree of knowledge in this important division of medical science.

